# Actively Coping with Violation: Exploring Upward Dissent Patterns in Functional, Dysfunctional, and Deserted Psychological Contract End States

**DOI:** 10.3389/fpsyg.2018.00054

**Published:** 2018-02-06

**Authors:** René Schalk, Melanie De Ruiter, Joost Van Loon, Evy Kuijpers, Tine Van Regenmortel

**Affiliations:** ^1^Tranzo, Tilburg University, Tilburg, Netherlands; ^2^HR Studies, Tilburg University, Tilburg, Netherlands; ^3^Economic and Management Sciences, North-West University, Potchefstroom, South Africa; ^4^Center for Leadership and Management Development, Nyenrode Business University, Breukelen, Netherlands; ^5^HIVA, KU Leuven, Leuven, Belgium

**Keywords:** psychological contract violation resolution, problem-focused coping, upward dissent, psychological contract end states, post-violation model

## Abstract

Recently, scholars have emphasized the importance of examining how employees cope with psychological contract violation and how the coping process contributes to psychological contract violation resolution and post-violation psychological contracts. Recent work points to the important role of problem-focused coping. Yet, to date, problem-focused coping strategies have not been conceptualized on a continuum from constructive to destructive strategies. Consequently, potential differences in the use of specific types of problem-focused coping strategies and the role these different strategies play in the violation resolution process has not been explored. In this study, we stress the importance of focusing on different types of problem-focused coping strategies. We explore how employee upward dissent strategies, conceptualized as different forms of problem-focused coping, contribute to violation resolution and post-violation psychological contracts. Two sources of data were used. In-depth interviews with supervisors of a Dutch car lease company provided 23 case descriptions of employee-supervisor interactions after a psychological contract violation. Moreover, a database with descriptions of Dutch court sentences provided eight case descriptions of employee-organization interactions following a perceived violation. Based on these data sources, we explored the pattern of upward dissent strategies employees used over time following a perceived violation. We distinguished between functional (thriving and reactivation), dysfunctional (impairment and dissolution) and deserted psychological contract end states and explored whether different dissent patterns over time differentially contributed to the dissent outcome (i.e., psychological contract end state). The results of our study showed that the use of problem-focused coping is not as straightforward as suggested by the post-violation model. While the post-violation model suggests that problem-focused coping will most likely contribute positively to violation resolution, we found that this also depends on the type of problem-focused coping strategy used. That is, more threatening forms of problem-focused coping (i.e., threatening resignation as a way to trigger one’s manager/organization to resolve the violation) mainly contributed to dysfunctional and deserted PC end states. Yet, in some instances the use of these types of active coping strategies also contributed to functional violation resolution. These findings have important implications for the literature on upward dissent strategies and psychological contract violation repair.

## Introduction

The psychological contract refers to an employee’s interpretation of the conditions underlying the exchange agreement between him or herself and the organization (Rousseau, 1989, 1995). The conditions underlying the psychological contract represent the inducements the employee feels that the organization has promised to deliver, such as bonuses, career advancement opportunities and challenging work, and the contributions the employee feels he or she is obliged to give back, such as loyalty, effort, and extra-role behavior (Conway and Briner, 2005; Ng and Feldman, 2012). When an organization does not uphold its end of the deal, employees may experience psychological contract breach and violation (Morrison and Robinson, 1997). Psychological contract breach refers to the “perception that the organization has failed to fulfill promised obligations” (Bordia et al., 2010, p. 1579), while psychological contract violation denotes the strong negative emotional responses such as anger, frustration, and distress that may follow from the perception of psychological contract breach (Morrison and Robinson, 1997; Bankins, 2015). According to Tomprou et al. (2015) negative emotional responses are particularly likely to occur following breaches of ‘important obligations’ and ‘losses significant to the employee’ (p. 1). Hence, Tomprou et al. (2015) conceptualize violation as “a highly significant breach that elicits strong negative emotional reactions” (p. 1). Since Tomprou et al. (2015) consider the circumstances in which breach results in violation, we follow this conceptualization.

Studies have shown that psychological contract violation is negatively related to outcomes such as job satisfaction (Callea et al., 2016), turnover intentions (e.g., Arshad, 2016; Salin and Notelaers, 2017), depressive mood states (Priesemuth and Taylor, 2016) and counterproductive behavior (Griep et al., 2016). Yet, there is not a lot of knowledge and understanding about the way in which employees cope with psychological contract violation and how the coping process leads to restoration of the psychological contract. To the best of our knowledge, three studies – one conceptual and two empirical – have considered coping processes and sense making in response to violation (Parzefall and Coyle-Shapiro, 2011; Bankins, 2015; Tomprou et al., 2015). Although these studies are very valuable to psychological contract theory and research, several important areas remain underexplored.

Tomprou et al. (2015) suggest that problem-focused coping is an important way in which employees can cope with psychological contract violation. Problem-focused coping encompasses actions that strive to eliminate an issue or reduce its effect (Tomprou et al., 2015; Hershcovis et al., 2017). Examples include “speaking up or acting to solve the problem and seeking advice or other instrumental aid” (Tomprou et al., 2015, p. 8). Hence, employee voice is considered a type of problem-focused coping (Tomprou et al., 2015). A qualitative study by Parzefall and Coyle-Shapiro (2011) provides support for the role of problem-focused coping, and voice in particular, in response to psychological contract violation. More specifically, when employees experienced strong emotional reactions in response to breach (i.e., violation feelings), they were likely to use voice, by speaking up in order to understand why the organization did not fulfill its commitments and to remedy the situation. Although this study provides some initial support for the use of problem-focused coping to eliminate or mitigate experiences of psychological contract violation, we contend that the *way* in which employees speak up also plays an important role in the contract repair process (De Ruiter et al., 2016).

The importance of the nature of communication has been highlighted previously in research on leader-member exchange as well. For example, Geertshuis et al. (2015) suggest that when communication between an employee and his or her manager is good-natured this is likely to result in positive outcomes for the employee, such as receiving relevant information and favorable performance reviews. Yet, when communication is perceived as unfriendly, the manager may be less willing to provide helpful information. This likely translates to the psychological contract repair process as well. For example, when employees speak up to their managers about violation in a more competent way, a manager will likely be more open to taking action which may positively influence the repair process, compared to instances in which employees speak up about violation in a less competent or unpleasant way (De Ruiter et al., 2016). However, current conceptualizations of problem-focused coping do not focus on how employees speak up to solve the issue nor do existing conceptualizations distinguish between a range of competent and less competent problem-focused coping strategies. In this study, we focus on a specific type of voice, namely employee upward dissent (Kassing, 2005), to address this gap in the literature.

An employee expresses dissent when he or she perceives differences between an existing and desired situation, and responds to this realization by protesting, complaining or disagreeing with the undesired situation (Kassing, 1997, 1998). Employee upward dissent refers to speaking up, disagreeing with or complaining about negative workplace situations to supervisors and management (Kassing, 2002). Employee upward dissent strategies have been conceptualized on a continuum ranging from competent to less competent strategies (Kassing, 2002, 2005; Kassing and Kava, 2013). By conceptualizing employee upward dissent strategies as types of problem-focused coping strategies, we are able to take into account the way in which employees voice dissatisfaction with violation, thereby contributing to the research on problem-focused coping strategies in response to psychological contract violation. Moreover, by focusing on a range of competent to less competent strategies, we are able to examine whether the way in which dissent is expressed differentially influences the violation repair process.

By focusing on employee upward dissent strategies, this study also provides a theoretical extension on the role of voice in response to psychological contract violation. According to De Ruiter (2017), justice-oriented or remedial voice is particularly relevant in the context of breach and violation. Justice-oriented voice takes place in response to transgressions or negative situations in the workplace (Klaas et al., 2012). This is also in accordance with earlier considerations of voice responses in the context of violation. More specifically, Rousseau (1995) explained that employees use voice in order to “remedy the violation” (p. 136). However, to date, studies have largely focused on proactive (e.g., Si et al., 2008) or destructive voice constructs (e.g., Ng et al., 2014). Proactive voice refers to voluntary behavior that benefits the organization (LePine and Van Dyne, 1998), while destructive voice refers to negative behavior aimed at damaging one’s employer (Ng et al., 2014). Although it is interesting to examine these outcomes in response to breach and violation, these concepts do not capture voice as originally intended by psychological contract scholars.

To the best of our knowledge, one study has considered justice-oriented voice in response to psychological contract breach (Turnley and Feldman, 1999). Hence, there is a lack of research on the role of speaking up about violation in an attempt to address the violation. Moreover, the conceptualization and operationalization of justice-oriented voice does not reflect how employees speak up to supervisors and managers. Scholars have identified several justice-oriented voice strategies, such as voicing concerns to one’s manager, open door policies and grievance procedures (Olson-Buchanan and Boswell, 2008; Klaas et al., 2012). Yet, current conceptualizations and measures of justice-oriented voice do not consider different ways in which ‘voicing concerns to one’s managers’ can be expressed. Yet, Rousseau (1995) indicated that voice in response to violation can take different forms, including speaking to managers and using threats (e.g., threats of resignation). Hence, a focus on different types of strategies that are used to speak up about violation is long overdue. By focusing on employee upward dissent strategies, we are better able to capture the different voice responses that can be used in response to violation (Rousseau, 1995) and are therefore better able to examine the role of voice in response to violation.

In this study, we draw from two types of qualitative data (interviews with managers and data from Dutch court cases) to understand different ways in which employees express dissent about psychological contract violation, the types of strategies that are used over time and the outcome (i.e., psychological contract end state) of the dissent process.

### Actively Coping with Violation: Employee Upward Dissent

Parzefall and Coyle-Shapiro (2011) conducted a qualitative study with the goal to understand the sense making process that takes place after psychological contract breach has occurred. Although these authors refer to psychological contract breach, the results of their study also reflect instances of psychological contract violation. To illustrate, some employees experienced instant emotional responses such as anger following a breach (Parzefall and Coyle-Shapiro, 2011), exemplifying instances of violation. Results of the study showed that employees who experienced such instant emotions in response to breach used voice to try to get the organization to explain its behavior and remedy the situation. Drawing from the results of this study, it can be concluded that when employees experience psychological contract violation, they may actively seek information and voice their concerns to organizational representatives in an attempt to address the situation. According to De Ruiter et al. (2016), employee upward dissent strategies capture voice responses to organizational representatives about unfulfilled obligations particularly well.

According to Kassing (1997, 1998), employees may express dissent to different audiences. Based on the type of audience to whom employees express dissent, Kassing (1997, 1998) distinguished between three types of dissent, upward, lateral, and displaced dissent. Upward dissent refers to voicing dissatisfaction or expressing disagreement with workplace situations to functional organizational players such as supervisors and management (Kassing, 2002). Lateral dissent occurs when an employee expresses dissent to co-workers, while displaced dissent refers to complaining or voicing dissatisfaction to non-work friends and family (Kassing, 2002). In this study, we specifically focus on upward dissent since this seems to most accurately reflect the active problem-focused coping responses to violation that were found in previous research. For example, Parzefall and Coyle-Shapiro (2011) found that employees spoke up in order to have “the employer to justify its behavior, to aid in finding a plausible explanation and to take corrective action” (p. 18). Since employees who experience violation were actively requesting the organization to resolve the situation, this seems to exemplify upward rather than lateral or displaced dissent. Rousseau et al. (1992 as cited in Rousseau, 1995) also found that employees targeted voice about violation to organization representatives including supervisors and senior management. More specifically, if employees would like the organization to change the situation, they will speak up to effectual audiences such as supervisors and management, rather than ineffectual audiences such as co-workers and non-work friends.

#### Employee Upward Dissent Strategies

Kassing (2002) described five types of employee upward dissent strategies, direct factual appeal, solution presentation, circumvention, threatening resignation, and repetition. Direct factual appeal deals with employees gaining support for their claims with factual information, which is accumulated through a combination of physical evidence and personal work experience (Kassing, 2009). Solution presentation deals with employees presenting a solution for their claims rather than or even in addition to evidence of the problem (Kassing, 2009). Circumvention refers to employees speaking up to someone above one’s direct supervisor in the organizational hierarchy (Kassing, 2009). Employees may employ circumvention for different reasons. An employee may go to someone higher in the chain of command when he or she believes that one’s direct manager is not willing or unable to respond to the employee’s dissent. Yet, an employee might also use circumvention when he or she wants to express dissent about potentially dubious behavior of one’s direct manager (Kassing, 2007). Threatening resignation refers to employees who warn that they are going to leave the organization if no appropriate action is taken (Kassing, 2009). Repetition refers to “repeated attempts to express dissent about a given topic at multiple points across time with the intention of eventually retaining receptivity to the dissent issue” (Kassing, 2002, pp 197–198). The repeated use of upward dissent is not limited to the same type of dissent strategy. To illustrate, employees may first express dissent through the use of direct factual appeal and solution presentation, while they may turn to other strategies such as circumvention and threatening resignation later on in the dissent process. According to Kassing (2009), employees are more likely to use direct factual appeal and solution presentation on multiple occasions, whereas circumvention and threatening resignation are not repeated as much. Repetition is different from the other four strategies since repetition refers to the use of (different) upward dissent strategies over time.

According to Kassing (2002, 2005) and Kassing and Kava (2013), upward dissent categories can be distinguished in more prosocial, competent forms (also face-preserving) and in less competent, more face-threatening forms of dissent. Direct factual appeal and solution presentation are considered more prosocial forms of dissent, circumvention and threatening resignation are considered more threatening forms of dissent, while repetition is somewhere in the middle (Kassing and Kava, 2013). The differences between face-preserving and face-threatening or competent and less competent strategies lies in the perception of the strategy’s competence, appropriateness, and effectiveness. Kassing and Kava (2013) point out that direct factual appeal and solution presentation are perceived as competent, while repetition is perceived as somewhat competent. According to Kassing and Kava (2013), circumvention and threatening resignation are considered less competent and more face-threatening. Garner (2016) found that managers are likely to perceive direct factual appeals as effective strategies and solution presentation as appropriate strategies, whereas managers’ perceptions of repetition were negatively related to appropriateness.

An important reason why some upward dissent strategies are considered more appropriate, competent and effective than other upward dissent strategies is the perceived level of threat (Kassing, 2005). While some strategies are more likely to preserve a manager’s status and indicate respect, other strategies indicate a lack of respect and disregard a manager’s superior position (Kassing, 2005; De Ruiter et al., 2016). To illustrate, solution presentation is considered a competent, face-preserving strategy because “employees protect a manager’s face because they do not hold a manager personally accountable and work together to resolve the breach of obligation” (De Ruiter et al., 2016, p. 196). Yet, circumvention is an incompetent, face-threatening strategy because there is a lack of regard for the manager’s position (an employee bypasses one’s manager and addresses the situation to the manager’s manager) and it can signal a lack of respect for one’s manager (by taking up the issue with the manager’s manager, the employee’s manager may be put in an awkward position with his/her own manager).

#### Expression of Dissent over Time

According to Kassing (2009), when employees first express their disagreement with workplace situations to their superiors, they are most likely to use a more prosocial, competent form of dissent (i.e., direct factual appeal and solution presentation). Kassing (2009) goes on to suggest that when employees have used such competent upward dissent strategies on multiple occasions without receiving the desired response, they will likely move to less competent, more face-threatening forms of upward dissent (i.e., circumvention and threatening resignation). The use of dissent strategies over time is similar to the strategy ‘repetition.’

### Upward Dissent As Problem-Focused Coping

According to Tomprou et al. (2015), the violation resolution process is affected by the type of coping strategy an employee uses in response to perceived psychological contract violation. Tomprou et al. (2015) suggest that problem-focused coping is likely to positively impact the resolution process. Yet, this assumption seems to be based on the premise that problem-focused coping strategies are constructive. More specifically, Tomprou et al. (2015) refer to adaptive or functional problem-focused coping strategies such as “acting to solve the problem” or active coping (Carver et al., 1989). Although the strategies outlined in Tomprou et al. (2015) represent aspects of problem-focused coping, they seem limited to constructive strategies. Yet, the literature on problem-focused coping also suggests that problem-focused coping strategies can be destructive.

Folkman et al. (1986) and Folkman and Lazarus (1988) identified confrontive coping as a more destructive form of problem-focused coping. This type of coping is characterized by anger and risk-taking and refers to “aggressive efforts to alter the situation” (Folkman et al., 1986, p. 995). Although more positive and less positive problem-focused coping strategies have been identified in the literature, we contend that conceptualizations of problem-focused coping strategies do not seem to exist on a continuum in which one type of coping strategy (e.g., speaking up to higher-ups about the negative situation in order to change it) can be expressed on a continuum from more to less constructive. Although Folkman et al. (1986) and Folkman and Lazarus (1988) identified two types of problem-focused coping, confrontive coping and planful-problem solving, wherein the former is hostile and interpersonal and the latter is more controlled and not interpersonal, the number of strategies is limited to two. Since a continuum of speaking up to resolve a stressful situation in a more competent and less competent way does not seem to exist in the literature on problem-focused coping, we draw from the literature on employee upward dissent to conceptualize problem-focused coping in response to psychological contract violation.

According to Kassing and Kava (2013), the five employee upward dissent strategies can be placed on a continuum from competent (face-preserving) to less competent (face-threatening) strategies. These strategies can also be linked to planful-problem solving and confrontive coping. To illustrate, coming up with solutions is part of planful-problem solving (Folkman et al., 1986), which can be linked to the upward dissent strategy ‘solution presentation,’ while trying something risky which is part of confrontive coping (Folkman et al., 1986) can be linked to ‘circumvention’ and ‘threatening resignation.’ Although the problem-focused coping strategies can be linked to upward dissent, the five upward dissent strategies offer a wider range of employee responses to violation than the two types of problem-focused coping. Therefore, by conceptualizing upward dissent strategies as problem-focused coping strategies this offers the opportunity to explore whether different types of problem-focused coping strategies can be considered beneficial in the violation resolution process or whether these strategies differentially affect the resolution process.

### Psychological Contract End States

Tomprou et al. (2015) indicate that following a violation of the psychological contract, the resolution process can result in four post-violation end states, namely psychological contract thriving, psychological contract reactivation, psychological contract impairment, and psychological contract dissolution. The first one, psychological contract thriving, is a highly functional end state. There is an improved relationship with one’s employer which is guided by a psychological contract that is more favorable than before the violation (Tomprou et al., 2015; Solinger et al., 2016). The second one, psychological contract reactivation is also a functional end state. In this situation, the content of the post-violation psychological contract corresponds to the content of the pre-violation contract (Tomprou et al., 2015; Solinger et al., 2016). Psychological contract impairment, the third end state identified by Tomprou et al. (2015), is a dysfunctional outcome. It refers to a situation that is less favorable than before the violation. Finally, Tomprou et al. (2015) refer to psychological contract dissolution. This is the most dysfunctional state and implies that employees remain in a ‘chronic state of violation’ (Tomprou et al., 2015).

The end states identified in the post-violation model are limited to employees who stay with their employer following a violation (Tomprou et al., 2015). Nevertheless, as Tomprou et al. (2015) also point out, employees who have experienced a violation of their psychological contract may also leave the organization. Consequently, we deemed it appropriate to also examine the active coping process of those employees who exit the organization after a perceived violation. There might be interesting differences in the dissent process of those who exit versus those who stay with their organizations. We therefore refer to a fifth psychological contract end state, namely desertion (Schalk and Roe, 2007). This end state refers to situations in which employees have exited the organization following a violation.

According to the post-violation model, problem-focused coping will likely result in functional psychological contract end states. Yet, Tomprou et al. (2015) have not distinguished between different problem-focused coping strategies. Therefore, it is important to explore if different problem-focused coping strategies (ranging from constructive to less constructive/destructive) similarly contribute to the resolution process or whether some may be more beneficial for violation resolution than others.

### Research Questions

In the previous sections, we first explored the role of coping processes in response to psychological contract violation and particularly focused on the role of problem-focused coping. Next, we highlighted the need to focus on a continuum of problem-focused coping strategies, rather than just examining constructive forms of problem-focused coping in relation to violation. We also explained the relevance of upward dissent following a psychological contract violation and provided arguments as to why upward dissent strategies can be conceptualized as different forms of problem-focused coping. Considering conceptual and empirical work on the perceived effectiveness and appropriateness of different upward dissent strategies, we suggest it is likely that some upward dissent strategies may be more beneficial to the violation resolution process than others. Moreover, based on existing research on the use of employee dissent strategies over time, it is likely that employees will first use competent, face-preserving strategies followed by less competent strategies over time. Regarding the violation resolution process, we distinguished between five psychological contract end states, ranging from functional (thriving and reactivation) to dysfunctional (impairment and dissolution) to desertion (exiting the organization). To explore the dissent process following a perceived violation and the role of this process in violation resolution more fully, we aimed to answer the following research questions:

Research question 1: Which upward dissent strategy was used first following a perceived violation?
Research question 2: How does the use of the first dissent strategy affect the outcome (i.e., psychological contract end state)?
Research question 3: How does the use of upward dissent strategies evolve over time?
Research question 4: How does the dissent process (i.e., the use of upward dissent strategies over time) affect the outcome (i.e., psychological contract end state)?

## Materials and Methods

Data that were used for this study were descriptions of sequences of events after psychological contract violation. The descriptions were derived from two sources. Twenty-three cases were based on in-depth interviews with managers and eight cases were derived from descriptions of Dutch court cases. The following four criteria were used to select the cases. First, cases needed to contain a description of psychological contract violation situations in the employee-organization relationship. Second, cases needed to include evidence of strong emotions involved. Third, cases needed to include a description of a dissent strategy or dissent strategies over time. Fourth, cases needed to include a description of the dissent outcome, which in this study refers to the psychological contract end state.

Based on the criteria described in the previous paragraph, 23 cases based on in-depth interviews were selected from a larger set of 35 interviews performed by the third author. Twelve interviews were not included because those interviews either did not represent examples of psychological contract violation or because no descriptions of employee upward dissent were provided. The interviews were conducted in one of the largest car-lease service providers in Netherlands. This organization was selected because the organization had recently gone through a series of mergers and acquisitions that resulted in substantial changes in organization culture, performance and reward systems, work environment, and work content. Therefore, it was likely that a large number of employees experienced a psychological contract violation. In total, 10 managerial-level employees participated in this research, five females and five males. The age of the respondents ranged between 21 and 50 years with an average of 34 years. Participants’ average tenure with the organization was 8.2 years and each of the participants was responsible for a department with an average of 10.5 employees. We opted to interview managers because of the following reasons. First, most studies on the evaluation of the psychological contract focus on the views of employees. The managerial perspective has been largely neglected. Second, since the focus of our study is on upward dissent, managers are a good source to provide information on their perception on how the employee dissents. More specifically, employees are very likely to use four out of five upward dissent strategies (direct factual appeal, solution presentation, repetition, and threatening resignation) with their manager. These dissent strategies are targeted at one’s direct manager, and therefore managers are in a good position to discuss the employee upward dissent process. The other upward dissent strategy, circumvention, refers to going above the direct manager’s head. Hence, a possible question might be whether managers are aware of instances in which their direct reports used this strategy. The data provided by the managers in this study showed that the managers were aware of instances in which circumvention was used. Therefore, managers are in a good position to discuss an employee’s use of upward dissent in response to psychological contract violation. Third, managers can also provide information on their own role in the process, and the ways in which they have tried to remedy the psychological contract violation.

In semi-structured interviews conducted by the third author, the interviewer first elaborated on the central concepts of the study, such as psychological contract breach and provided background information on the study. This information was provided to help the participating managers think about cases in which employees experienced a breach in their psychological contract, employee emotions that were involved in these situations (to determine whether experiences of violation had occurred) and which types of employee active responses managers observed after psychological contract violation. For each case provided, interviewees were asked to answer questions about (1) the breach itself, (2) employee emotions in response to breach – this was necessary in order to determine whether employees had in fact experienced psychological contract violation, (3) the employee’s active behavior in response to the violation (these questions were aimed at learning about whether or not and which upward dissent strategies were used), (4) how the employee’s active responses to violation evolved over time, and (5) finally the state of the relationship/contract after this process. Based on the answers that were given to the questions, probing and additional sub-questions were used.

The interviews were conducted in May 2013 and each interview lasted between 40 and 60 min. At the beginning of each interview, the participants were assured that the interview was strictly confidential and that publication of the research would not reveal the participant’s identity or the identity of their organization. All participants gave permission to audio-record the interview.

Eight cases were derived from an electronic, open access database containing information on court cases in Netherlands^1^, covering the period 1999 until 2017. The database includes anonymous descriptions of the reports made by the court. Each case includes a description of the problem or conflict, the views of the parties involved, and the argumentation of the court to come to a verdict. In the Dutch database, not all court cases are included^2^. We searched the databases using the following search terms: ‘Conflict,’ ‘employee,’ ‘manager,’ ‘broken trust,’ ‘promise,’ ‘end of contract,’ and ‘trust.’ By employing these search terms, we ensured that the selection of cases would be limited to employment relationships. The search provided a list of 518 court cases that fulfilled the search criteria. The court cases were read and selected for coding when the case met the criteria outlined above. Eight cases fulfilled the criteria. The cases involved seven male and one female employee. The ages ranged between 30 and 54 years with an average of 46.7 years (the age of one respondent was unknown). The job tenure ranged between 3 and 23 years. Moreover, the occupation of the respondents was very diverse, e.g., financial worker, teacher, business analyst, and a nurse.

Although the perspectives and the PC end states in the two sources of data are partly different (manager perspective in the interviews, outsider perspective in the court cases, interviews have different end states, court cases are limited to one end state), all cases contain descriptions of observations of a situation of psychological contract violation, subsequent employee dissent behaviors from a non-employee perspective and descriptions of PC end states. Moreover, the coding strategy that was applied for analyzing the interview cases and the court cases was the same. Consequently, we decided to group the 23 manager descriptions and the eight court cases together. In total, our sample thus consisted of 31 cases describing upward dissent in response to psychological contract violation. With regard to repetition of upward dissent strategies (i.e., upward dissent shifts), 24 cases were analyzed (six manager cases and one court case were excluded since these cases only described the use of one dissent strategy at one point in time).

### Coding Strategy

A coding framework, consisting mainly of theoretically-driven codes, was used to label the descriptions in the cases. In the following sections, for each of the main themes, the theoretically-driven codes are outlined and examples are provided to clarify the coding process. In addition to theoretically-driven codes, four codes were derived from the data to code the different types of repetition. These data-driven codes are explained in the section on upward dissent strategies.

### Upward Dissent Strategies

Kassing’s (2002) five upward dissent categories (direct factual appeal, solution presentation, repetition, circumvention, and threatening resignation) were used to code descriptions of active employee responses following psychological contract violation. The coding of the strategies direct factual appeal, solution presentation, circumvention, and threatening resignation was fairly straightforward. When the description of the employee’s response fit the conceptualization of one of these four dissent strategies, it was coded as such. Repetition is somewhat different from the other four strategies; it occurs when dissent strategies are used on multiple occasions about the same dissent issue. Hence, the labeling of this type of dissent deserves some more attention. We examined repetition by examining dissent shifts. Yet, the conceptualization of repetition is quite broad. According to Kassing (2002), repetition occurs when an employee employs dissent about the same issue on multiple occasions. This means that employees can repeat competent dissent strategies, but they may also shift in their use of competent and less competent strategies. Since the literature on dissent has not distinguished between different types of repetition, data-driven codes were derived based on four different types of repetition found. Upward repetition refers to the use of more competent to less competent strategies over time. Downward repetition refers to the use of less competent to more competent strategies over time. Mixed repetition refers to the use of competent and less competent strategies interchangeably. Finally, persistent repetition refers to the use of the same dissent strategy over time.

### Psychological Contract End States

The coding of the psychological contract end states was based on the four end states (thriving, reactivation, impairment, and dissolution) described by Tomprou et al. (2015) supplemented by the end state ‘desertion’ as described by Schalk and Roe (2007). Based on the initial coding of the data, it was decided to create three higher-order codes. That is, there were not enough cases for some of the end states (particularly with regard to thriving and dissolution) to warrant analysis at this level. Consequently, it was decided to distinguish between those end states that were described in the literature as more functional (i.e., thriving and reactivation), those that were described as dysfunctional (i.e., impairment and dissolution) and those that referred to exiting the organization (i.e., desertion).

### Analysis Strategy

To analyze the use of upward dissent strategies over time (i.e., different forms of repetition) we used a logic-model analysis (Yin, 2014). A logic model “stipulates and operationalizes a complex chain of occurrences or events over an extended period of time” in which the events are staged in repeated cause-effect-cause-effect patterns (Yin, 2014, p. 155). The analysis consists of matching empirically observed events (e.g., dissent behaviors) to theoretically predicted events, in the case of changes in dissent behaviors over time. The logic model is related to pattern matching. Due to the sequential stages, however, logic models can be distinguished as a separate analysis technique (Yin, 2014, p. 155). Logic models can be applied on small samples. Samuel et al. (2015) used this method to analyze changes associated with injuries in six athletes.

We applied an individual-level logic model of each case, which was considered a unit of analysis with the purpose of examining similar or dissimilar change processes over time. The logic-models for ‘functional psychological contract end states,’ ‘dysfunctional psychological contract end states,’ and ‘deserted psychological contracts’ were compared.

## Results

Prior to presenting the results of the analyses, it is important to provide some contextual information regarding the cases retained for the study. It is important to note that all instances of breach described by the managers were accompanied by strong emotions such as anger and frustrations on the part of the employee. Since the breaches were accompanied by such strong emotional reactions, the cases described fit the conceptualization of psychological contract violation as presented in Tomprou et al. (2015). **Table 1** provides a short overview of the characteristics of the cases. The type of psychological contract violation is indicated. In addition, the first dissent strategy, the type of repetition (dissent shift), and the PC end states are provided in the table. Moreover, information on the time frame (for those cases for which it was available) is presented. With regard to the time frame, it can be concluded that for those situations in which only one dissent strategy was used, the dissent process took less long than for those situations in which repetition was used.

**Table 1:** Details cases.

No.	Data code	Description psychological contract violation	First dissent strategy	Repetition (dissent shift)	Time	End state
1	m1e1	Promise to provide a permanent contract was broken	Direct factual appeal	Yes, mixed	Unknown	Functional
2	m1e2	Insufficient supervisory supportive behavior	Direct factual appeal	Yes, mixed	About a half year	Functional
3	m1e3	Excellent performance not rewarded	Direct factual appeal	Yes, mixed	A couple of months	Functional
4	m2e2	Pay raise not granted	Direct factual appeal	No	A few weeks	Functional
5	m2e3	Performance evaluation below expectations	Solution presentation	Yes, upward	Unknown	Functional
6	m2e4	Conflict with external business partner	Direct factual appeal	Yes, upward	Unknown	Functional
7	m3e2	Difficulties with transition to other position/firm	Solution presentation	Yes, upward	A couple of weeks	Functional
8	m3e3	Performance evaluation below expectations	Direct factual appeal	Yes, upward	A short period of time	Functional
9	m4e1	Additional financial compensation decreases	Direct factual appeal	Yes, mixed	A few years	Functional
10	m4e2	Lack of support from IT department	Solution presentation	No	Unknown	Dysfunctional
11	m5e1	No pay raise with change to other position	Direct factual appeal	No	Unknown	Functional
12	m5e3	Accused of negative behavior toward colleague	Circumvention	Yes, upward	Unknown	Dysfunctional
13	m6e1	Promotion denied	Threatening resignation	Yes, downward	Unknown	Dysfunctional
14	m6e2	Pay raise not granted	Direct factual appeal	No	One month	Dysfunctional
15	m7e1	Change to performance based appraisal system	Direct factual appeal	Yes, upward	A few months	Functional
16	m8e1	Performance after promotion insufficient	Threatening resignation	No	A few months	Dysfunctional
17	m9e1	Change of work days not granted	Circumvention	Yes, upward	Unknown	Dysfunctional
18	m10e2	Performance evaluation below expectations	Direct factual appeal	Yes, persistent	Unknown	Functional
19	m2e1	Differences in expected work behavior	Solution presentation	Yes, mixed	A few weeks	Desertion
20	m3e4	Difficulties with transition to other position/firm	Direct factual appeal	Yes, upward	Unknown	Desertion
21	m5e4	Promotion denied	Direct factual appeal	No	A long period of time	Desertion
22	m4e3	Difficulties with transition to other position/firm	Circumvention	Yes, upward	A few months	Desertion
23	m10e1	Difficulties with transition to other position/firm	Direct factual appeal	Yes, upward	A few months	Desertion
24	r1	Promised coaching in new position not provided	Direct factual appeal	Yes, mixed	One year	Desertion
25	r2	Promised open dialog in new position not provided	Circumvention	Yes, mixed	Three months	Desertion
26	r3	Promise to provide permanent contract was broken	Direct factual appeal	Yes, upward	Five months	Desertion
27	r4	Difficulties with transition to other position/firm	Direct factual appeal	Yes, upward	Seven months	Desertion
28	r5	No future perspectives provided	Direct factual appeal	No	Seven months	Desertion
29	r6	Absence for taking care of partner denied	Direct factual appeal	Yes, persistent	Four months	Desertion
30	r7	Promotion denied	Direct factual appeal	Yes, upward	Two years and four months	Desertion
31	r8	Performance evaluated as below standard	Direct factual appeal	Yes, mixed	Nine months	Desertion

### First Upward Dissent Strategy Used

Based on the analyses of the 31 cases, it was found that the majority of employees chose to first use a prosocial form of upward dissent. That is, 25 employees either used direct factual appeal or solution presentation in their initial dissent about violation. The majority of these employees (21 employees) used direct factual appeal, while fewer (four employees) used solution presentation. By using direct factual appeal, employees tried to convince the organization to revise the decision that led to a psychological contract violation. One employee, for example, was involved in a conflict with an external service partner and felt that her psychological contract was violated because the organization did not support her. She tried to persuade the supervisor that she was right and get the supervisor on her side by presenting facts and describing situations in which the external service partner acted in a negative way. Another employee, who experienced a violation because she did not get an extra bonus after a performance evaluation, which in her opinion had to be granted, created an appendix to the performance evaluation with notes on her perception of her own performance, which she wanted to be included in her performance appraisal. Another example is that of an employee who after he received a lower performance appraisal grade than he expected to be entitled to, presented all kinds of facts on his performance to try to change his performance grade.

In addition to the more prosocial forms of upward dissent, some employees chose to use a more threatening form when first employing an upward dissent strategy, including circumvention (four employees) and threatening resignation (two employees). An example of the former is an employee who went to the HR-manager and the manager of his manager to get support for his position that he should have been appraised with a better grade. An example of the latter is an employee who indicated that she would resign because she could not work in a team that accused her of behaving in a negative way (gossiping).

Next, we explored whether there were differences in the choice of the first upward dissent strategy used and the dissent outcome, which in this study refers to the PC end states (functional, dysfunctional, and desertion). Based on the analyses of the 31 situations, we found that 12 situations resulted in functional PC end states, six situations resulted in dysfunctional PC end states, and 13 situations ended in desertion. With regard to functional PC end states, we found that all employees decided to first use a prosocial form of upward dissent. That is, in 10 cases direct factual appeal was used first, whereas in two situations solution presentation was first employed. With regard to deserted PCs, employees also mostly began with prosocial forms of dissent. That is, 10 employees first used direct factual appeal, one first employed solution presentation, whereas two employees used circumvention – a more threatening form of dissent. In relation to dysfunctional PC end states, it was found that the majority (four employees) began with more threatening forms of dissent, while two employees used a more prosocial form of dissent.

### Shifts in the Use of Dissent Strategies over Time

In addition to examining the type of dissent strategy that was first used by employees, we were interested in exploring whether the use of upward dissent strategies shifted from more prosocial to more threatening forms of upward dissent over time. Our analyses of the cases were limited to those cases that included the use of at least two upward dissent strategies. Therefore, our sample size for these analyses was limited to 24 cases. Of these 24 cases, 10 cases resulted in functional PC end states, three cases resulted in dysfunctional PC end states and 11 cases resulted in deserted PCs.

The results showed that in slightly more than half of the cases (13, 54%) there was an upward shift (i.e., upward repetition) in the use of dissent strategies. This means that the use of dissent strategy shifted up along the continuum from more prosocial to more threatening. In eight instances (33%), the use of dissent strategies was mixed (mixed repetition). This means employees shifted back and forth between more prosocial and more threatening dissent strategies. In the remaining three cases, one situation was exemplified by a downward shift (downward repetition; shifting from a more threatening to a more prosocial form of upward dissent) and two situations were characterized by repetition of a prosocial upward dissent strategy (persistent repetition).

**FIGURE 1:**
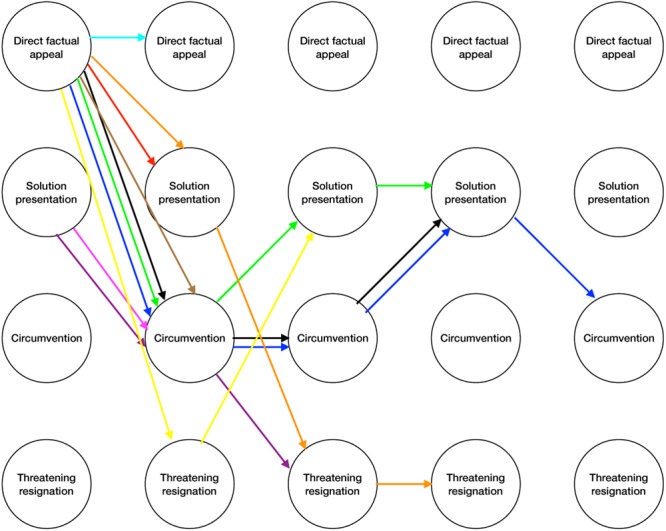
Logic model analyses functional psychological contract end states.

**FIGURE 2:**
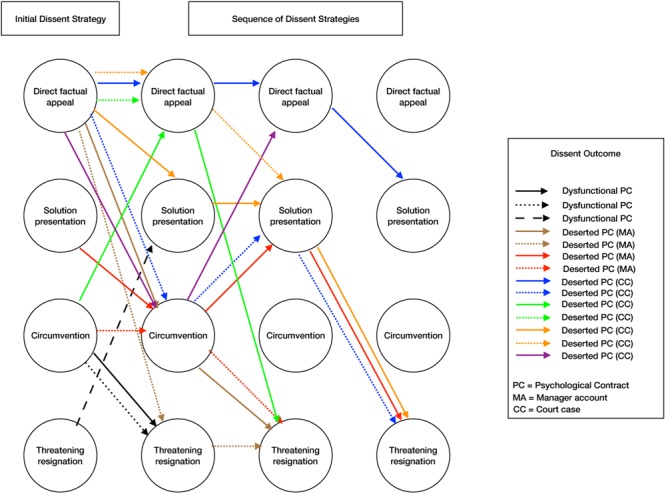
Logic model analyses dysfunctional and deserted psychological contract end states.

Next, we explored whether we could find any interesting differences in the dissent patterns over time in relation to the dissent outcome, i.e., the PC end state. We found that for all three PC end states, an upward shift in the use of upward dissent strategies was most often used. Half of the employees in the functional PC end state, shifted their use of dissent strategies up the continuum from more prosocial to more threatening types of upward dissent. In the dysfunctional PC end state, this was the case for two out of three employees, and in the deserted PC end state, this was true for 6 out of 11 employees. Yet, what is interesting to note is that with regard to functional PC end states, these employees always began with a prosocial form of upward dissent (direct factual appeal or solution presentation), whereas for the dysfunctional PC end state, employees began with a threatening form of upward dissent (circumvention) followed by a more threatening form of upward dissent (threatening resignation). Thus, despite the fact that in both instances employees most often displayed upward shifts in their use of dissent, the place along the continuum where employees began differed. The dissent patterns for the end states are depicted in **Figures 1, 2**. **Figure 1** presents the dissent patterns for functional PC end states, whereas **Figure 2** represents the dissent patterns for the negative end states. In **Figure 2**, we included the dissent patterns for both the dysfunctional and deserted PC end states.

## Discussion

The aim of this study was to explore how employees actively cope with psychological contract violation. By focusing on upward dissent strategies, we aimed to examine how the use of different types of problem-focused coping contributed to the violation resolution process. Our first two research questions addressed which upward dissent strategy was used first following a perceived violation and how the first strategy used influenced the dissent outcome, i.e., the psychological contract end state. We found that the majority of employees chose to first use a prosocial form of upward dissent, while some employees chose to use a more threatening form. When dissent resulted in functional PC end states, all employees had first used a prosocial form of upward dissent. When dissent resulted in dysfunctional PC end states, the majority had begun with more threatening forms of dissent, while a minority had used a more prosocial form of dissent. When the dissent process led to deserted PCs, employees had mostly begun with prosocial forms of dissent.

Regarding our third and fourth research question, we found that the dissent process evolved differently over time in relation to the dissent outcome. We found that for all three PC end states, upward repetition was most often used. In functional PC end states, employees always began with a prosocial form of upward dissent (direct factual appeal or solution presentation), whereas in the dysfunctional PC end state, employees began with a threatening form of upward dissent (circumvention) followed by a more threatening form of upward dissent (threatening resignation). Thus, despite the fact that in both instances employees most often displayed upward repetition in their use of dissent, the place along the continuum where employees began may have affected the dissent outcome.

Our study makes a number of contributions to the literature. According to Kassing (2009), employees are likely to first use a competent, prosocial form of dissent. Although our study largely supports this position for two psychological contract end states (functional and deserted), employees who were left with dysfunctional psychological contracts often chose to first use a more face-threatening form of dissent. Hence, our study indicates the importance of focusing on the dissent outcome (psychological contract end states in the present study) when examining the use of upward dissent strategies. Through our study we were able to link the initial use of more face-threatening dissent strategies to the psychological contract end state. Yet, it might also be that certain events before the violation (for example, violations having occurred one after the other in a short period of time) or the importance of the loss of inducement resulted in an employee’s choice to first use a face-threatening form of dissent.

Kassing (2009) suggests that it is likely that employees follow an upward shift in dissent strategies – which we have referred to as ‘upward repetition’ in our study. This means that employees are likely to first use prosocial, competent forms of dissent, and after having repeated these prosocial forms on several occasions move to more threatening forms of dissent. Our data only partly support this claim. That is, while the majority of employees shifted up along the prosocial – face-threatening dissent continuum, there were also employees who began with a face-threatening form (circumvention) followed by a more face-threatening form (threatening resignation), while other employees followed a mixed shift, switching back and forth between more prosocial and more face-threatening strategies (mixed repetition). The sequence of dissent strategies seemed to at least partly affect the psychological contract end state. Hence, future research should further examine the upward dissent strategy ‘repetition.’ The results of our study show that this strategy can be employed in different ways. Moreover, the use of different forms of repetition may likely differently affect the dissent outcome. It is important to further examine this.

Kassing (2005) found that employees perceive threatening resignation as the least competent dissent strategy. The results of our study seem to support this finding. That is, in the functional psychological contract end states, most employees did not resort to threatening resignation, yet this strategy was used by most employees in the dysfunctional and deserted psychological contract end states. Hence, our findings seem to suggest that the use of threatening resignation has at least partly contributed to these negative outcomes. Yet, more systematic and empirical research is needed to verify this proposition. Additionally, there were also some cases of functional psychological contract end states in which these threatening forms of dissent were used. Hence, the use of threatening resignation does not necessarily lead to negative outcomes. It is important to explore why threatening resignation may be effective for restoring violation in some situations but not in others. According to De Ruiter et al. (2016) this might be related to the supervisor’s relationship with one’s own supervisor. That is, if the supervisor has a good relationship with one’s manager, (s)he might be able to get more done for the employee in terms of resolving the situation. Another reason might be the employee’s performance. That is, managers (or other organization representatives) might be more willing to respond to threats of resignation for their highly performing employees versus employees who perform average.

Our study indicates that employees use various strategies to cope with psychological contract violation. According to the post-violation model (Tomprou et al., 2015), problem-focused coping is generally likely to have a positive effect on psychological contract resolution. Although Tomprou et al. (2015) have acknowledged that problem-focused coping might fail, they seem to link this to organizational situations (such as unsafe environments that punish those who speak up) and not to the type of problem-focused coping strategy used. Yet, the results of our study show that it is also important to consider the type of problem-focused coping strategy used. That is, the use of threatening forms of problem-focused coping (i.e., threatening resignation) seemed related to deserted and dysfunctional psychological contract outcomes, whereas it was less often linked to functional end states. Moreover, the use of competent problem-focused coping strategies can also negatively influence the resolution process and lead to dysfunctional and deserted psychological contracts. That is, some of the court cases revealed that even when employees had repeatedly used prosocial, competent forms of dissent (e.g., direct factual appeal followed by direct factual appeal followed by solution presentation) this could still result in deserted psychological contracts. Consequently, it is important to explore why the use of certain upward dissent strategies can be effective for resolving some situations while exacerbating others.

Tomprou et al. (2015) distinguish two feedback loops in their post-violation model. First, there is the discrepancy feedback loop, which signals a potential deviation between the current situation and the schema of mutual obligations. According to Tomprou et al. (2015), the decision of an employee to act after a discrepancy (for example by talking with one’s supervisor about the violation) is affected by the individual’s assessment of the likelihood that the discrepancy can be reduced. Our results extend the post-violation model on this feedback loop by showing that employees have multiple ways of reacting. The feedback that employees get on the results of the first way of dissenting tells them whether the discrepancy is resolved or not. When the discrepancy is not resolved, they will make a choice for follow-up behavior that according to them has the greatest likelihood of success. This can be either persisting in the same dissent strategy (persistent repetition) or choosing another one (for example through upward, downward, or mixed repetition). The meta-monitoring loop is the second feedback system distinguished by Tomprou et al. (2015). This feedback loop is associated with the persistence of attempts at discrepancy reduction and emotional recovery and influences the speed of discrepancy reduction. Our data did not enable us to analyze the speed of the discrepancy reduction, unfortunately.

### Limitations and Future Research Recommendations

Although this study makes some interesting contributions to the existing literature on coping with psychological contract violation and the violation restoration process, this study also has several limitations. First, the focus of this study was limited to problem-focused coping. Our study did not consider how other forms of coping (such as lateral dissent which is related to emotion-focused coping strategies like venting, and displaced dissent which is related to disengagement, Kassing, 2011) might be involved in coping with violation and the resolution process. The data revealed instances in which employees used lateral dissent. Yet, since this was not part of our study’s focus, these topics were not further explored during the interviews. Nevertheless, since lateral dissent (venting to co-workers) was mentioned in several cases, it is likely that employees do not only choose to use problem-focused coping but may, in response to violation, employ not only different forms of problem-focused coping, but also different forms of emotion-focused coping. This an important area that needs to be further explored.

Second, although we thoroughly analyzed the process of dissent over time after psychological contract violation, we have no full information on how previous events, the source and cause of the violation, as well as individual characteristics of the employee might have influenced the process. A past history of previous psychological contract violations might make an employee less likely to start with prosocial forms of dissent. Since there are multiple actors involved in psychological contracts (Alcover et al., 2017), the dissent strategy might be influenced by who or what the organizational agent is that caused the psychological contract violation. In the same vein, whether the violation was avoidable or inevitable is likely to play a role. Next to these issues associated with the organizational side, on the employee side, personality and self-esteem are likely to influence the choice of different types of coping (e.g., McCrae and Costa, 1986; O’Brien and DeLongis, 1996). This is an important area that needs to be further explored.

Third, although we were able to explore how the use of upward dissent strategies evolved over time, our data collection method did not allow us to inquire about the exact amount of time that was in between the use of different dissent strategies. We suggest that researchers employ daily or weekly diary studies to learn more about exact time periods between the use of dissent strategies. By employing diary studies, scholars are also able to examine the role of ‘resolution velocity’ (Tomprou et al., 2015, p. 563).

Fourth, we examined the role of upward dissent from the perspective of others than employees. In future research, it is important to examine the violation resolution process from both the perspective of the organization and that of the employee. Both parties may have different perceptions of the violation, its causes and effects. Considering both sides will provide insight in the degree of mutuality and can also highlight the role of reciprocity in the employment relationship.

Fifth, although we were able to explore the evolvement of dissent strategies in 24 cases, more empirical evidence is needed. That is, due to a smaller number of cases that exemplified end states such as thriving and dissolution, we were forced to group the categories thriving and reactivation, and impairment and dissolution together. Yet, the dissent process might be somewhat different when distinguishing between the four end states rather than two higher order categories. With a greater sample size, more different patterns in dissent behaviors for the different dissent end states might have been identified. Although the number of typical patterns in dissent is limited to four (upward, downward, mixed, and persistent), the number of possible combinations with three steps of four types of dissent behaviors is 64. With a larger sample size, a more fine-grained analysis of patterns might be possible. Finally, there can be other factors that can have an influence on the dissent process such as cultural differences, organizational culture, size of the organization and/or type of organization. Whether an organization can be characterized as an empowering organization (Peterson and Zimmerman, 2004) or not is likely to influence dissent behaviors of individual. In a cross-national study, cultural differences could be taken into account.

### Practical Implications

The results of this study show that employees are more likely to use more threatening forms of dissent behavior when their psychological contract violation is not restored (i.e., dysfunctional PC end states) or when they leave the organization (i.e., deserted PCs), and are likely to shift to the most threatening dissent strategy (i.e., threatening resignation). Therefore, it is of great importance that managers recognize early employee dissent behavior, consequently trying to stop the dissent process and restore the psychological contract violation prior to the escalation of the use of employee dissent.

Most employees start the dissent process in a constructive way. It is later in the process that a shift to more negative dissent behaviors can occur. It is important that managers reward the positive approach of the employees and seriously consider the opinions and feelings of the employee. The role and opportunities for the manager to remedy the violation should be clearly outlined. The role of the manager will be different in case (s)he has the opportunity to remedy the violation or help/support the employee in his/her attempts to find a solution versus cases where the manager can play no role in this respect.

## Ethics Statement

All subjects of the interviews gave written informed consent.

## Author Contributions

All authors listed have made a substantial, direct and intellectual contribution to the work, and approved it for publication.

## Conflict of Interest Statement

The authors declare that the research was conducted in the absence of any commercial or financial relationships that could be construed as a potential conflict of interest.

## Footnotes

1 https://uitspraken.rechtspraak.nl

2 For selection criteria see: https://www.rechtspraak.nl/Uitspraken-en-nieuws/Uitspraken/Paginas/Selectiecriteria.aspx
